# Down-regulation of *NCED* leads to the accumulation of carotenoids in the flesh of F_1_ generation of peach hybrid

**DOI:** 10.3389/fpls.2022.1055779

**Published:** 2022-11-03

**Authors:** Haiyan Song, Junhong Liu, Chaoqun Chen, Yao Zhang, Wenjing Tang, Wenlong Yang, Hongxu Chen, Mengyao Li, Guoliang Jiang, Shuxia Sun, Jing Li, Meiyan Tu, Lingli Wang, Zihong Xu, Ronggao Gong, Dong Chen

**Affiliations:** ^1^ College of Horticulture, Sichuan Agricultural University, Chengdu, China; ^2^ Horticulture Research Institute, Sichuan Academy of Agricultural Sciences, Chengdu, China; ^3^ Key Laboratory of Horticultural Crop Biology and Germplasm Creation in Southwestern China, Ministry of Agriculture and Rural Affairs, Chengdu, China

**Keywords:** peach, flesh color, carotenoids, NCED, transcriptome, metabolome

## Abstract

Flesh color is an important target trait in peach [*Prunus persica* (L.) Batsch] breeding. In this study, two white-fleshed peach cultivars were crossed [Changsong Whitepeach (WP-1) × ‘Xiacui’], and their hybrid F_1_ generation showed color segregation of white flesh (BF1) and yellow flesh (HF1). Metabolome analysis revealed that the flesh color segregation in the hybrid F_1_ generation was related to the carotenoid content. The decrease in β-carotene and β-cryptoxanthin in BF1 flesh and increase in β-cryptoxanthin oleate, rubixanthin caprate, rubixanthin laurate and zeaxanthin dipalmitate in HF1 flesh contributed to their difference in carotenoid accumulation. Transcriptome analysis demonstrated that compared with BF1, HF1 showed significant up-regulation and down-regulation of *ZEP* and *CCD8* at the core-hardening stage, respectively, while significant down-regulation of *NCED* in the whole fruit development stage. The down-regulation of *NCED* might inhibit the breakdown of the violaxanthin and its upstream substances and further promote the accumulation of carotenoids, resulting in yellow flesh. Therefore, *NCED* may be a key gene controlling the fruit color traits of peach. In this study, targeted metabolomics and transcriptomics were used to jointly explore the mechanism controlling the fruit color of peach, which may help to identify the key genes for the differences in carotenoid accumulation and provide a reference for the breeding of yellow-fleshed peach.

## Introduction

Peach [*Prunus persica* (L) Batsch], which belongs to the Rosaceae family, is native to China and has a long history of cultivation (Cao et al., 2021; Kalluri et al., 2022). Color is one of the most important sensory evaluation indicators of peach fruit quality, and has great impacts on its commercial value (Williamson et al., 2006). Peach flesh can be divided into white flesh, red flesh, yellow flesh and green flesh according to the color (Shen et al., 2012). White-fleshed peach is a common fresh eating variety in China. However, red-fleshed peach has gradually attracted more attention of consumers in recent years, and yellow-fleshed peach is particularly favored by European and American consumers. Yellow-fleshed peach contains abundant carotenoids, and has higher nutritional value than white-fleshed peach and better flavor than red-fleshed peach (Falchi et al., 2013).

The flesh color of peach is closely related to the type and content of pigments in the flesh, and the flesh color genes regulate the phenotype by regulating the synthesis, transport and accumulation of pigments (Gil et al., 2002). Chlorophyll, carotenoids and anthocyanins are main compounds determining the flesh color of fruits (Liu et al., 2022; Wu et al., 2022). White-fleshed peach has less pigment; and carotenoids and anthocyanins are the main pigments of yellow-fleshed and red-fleshed peach, respectively (Bai et al., 2016; Liu et al., 2019; Zhu et al., 2020). Compared with white-fleshed peach, yellow-fleshed peach contains a higher level of carotene. During growth and development, the total amount of carotenoids increases in yellow-fleshed peach while decreases in white-fleshed peach (Brandi et al., 2011).

Metabolic regulation of carotenoids in plants is diverse and complex, mainly involving the regulation of carotenoid synthesis and degradation. In the anabolism of carotenoids, the expression of genes in relevant anabolic pathways affects the synthesis of carotenoids. *PSY* gene encodes a phytoene synthase and is a key regulatory gene in the carotenoid synthesis pathway. High expression of PSY in tomato leads to the accumulation of carotenoids (Cao et al., 2019). Moreover, induced mutation of tomato *PSY* allele revealed that *PSY* is the only gene that promotes carotenoid synthesis during fruit development (Gady et al., 2012). In the carotenoid biosynthesis pathway, *PDS* plays a central regulatory role in processing colorless hydrocarbon phytoene to generate colored carotenoids. An experiment to inhibit the expression of *PDS* gene demonstrated that the content of carotenoids is positively correlated with the expression level of *PDS* (Qin et al., 2007). In addition, multiple ripening factors can regulate *ZDS* expression to influence carotenoid accumulation in tomato (McQuinn et al., 2020). LCYB and LCYE can catalyze lycopene to control the content of downstream metabolites, and their expression can regulate the carotenoid content in strawberry, apple, persimmon and other fruits (Ampomah-Dwamenasup et al., 2012; Zhu et al., 2015; Qi et al., 2019).

The color of plant tissues is not only related to carotenoid synthesis genes, but also affected by carotenoid degradation genes. For example, CCD4-catalyzed degradation of carotenoids affects the color of fruit flesh and flower. Several studies have demonstrated that inactivation or decreasing the expression of *CCD4* could result in the accumulation of carotenoids in peach flesh (Brandi et al., 2011; Adami et al., 2013; Falchi et al., 2013; Ma et al., 2014; Bai et al., 2016; Wen et al., 2020). *NCED* has been isolated from a variety of species, and it lower expression level can impede the breakdown of carotenoids, leading to changes in plant color (Rodrigo et al., 2006; Jiang et al., 2019; Lana et al., 2020; Yungyuen et al., 2021). Besides, NCED is also involved in ABA synthesis, thereby affecting peach fruit ripening and senescence (Wang et al., 2020). There has been no evidence proving that the expression of *NCED* affects the color of peach flesh.

Although there has been plenty of research on peach flesh color, the molecular mechanism for peach flesh color remains elusive. In this study, two white-fleshed peach cultivars, Changsong Whitepeach (WP-1) and ‘Xiacui’ (XC), were crossed to construct a hybrid F_1_ generation population. The fruit of the hybrid F_1_ generation showed segregation of flesh color into yellow and white, which could be ascribed to the difference in carotenoid accumulation in the flesh. This study combined targeted metabolomics and transcriptomic analysis to clarify the molecular mechanism for flesh color in peach hybrids.

## Materials and methods

### Plant materials and growth conditions

In our previous study, Changsong Whitepeach (WP-1) and ‘Xia Cui’ (XC) were used as hybrid parents to be crossed to construct a hybrid F_1_ generation population with 20 white-fleshed plants and four yellow-fleshed plants. We selected all the four yellow-fleshed F_1_ plants (HF1) and four white-fleshed F1 plants (BF1) with similar maturity and growth status for sample mixing, respectively. These two cultivars and their hybrid F_1_ generation were provided by the Horticultural Research Institute of Sichuan Academy of Agricultural Sciences, and cultivated in Jinxiu Dongshan Modern Agriculture Boutique Park (E 104°, N 30°), Yongxing Street, Tianfu New District, Chengdu. Fruit samples were collected at the core-hardening stage (S1, 78 days after pollination), color-transformation stage (S2, 94 days after pollination) and the mature stage (S3, 108 days after pollination). The fruit were peeled, cut into small pieces, quickly frozen in liquid nitrogen, and then stored in a ultra-low temperature refrigerator. Three biological replicates were set for each sample, and ten fruit were selected from each replicate.

### Determination of anthocyanin content

Determination of anthocyanin content in peach flesh was conducted according to the method described in a previous study (Xu et al., 2015). About 0.5 g of sample was taken, followed by the addition of 5 mL 1% hydrochloric acid-methanol solution. The sample was then put in a 15-mL centrifuge tube, shaken well, placed a dark place at 4°C for more than 20 h, and ultrasonicated for 30 min. After 5 min of centrifugation at 4°C and 8000 rpm, the absorbance was measured at 530 nm, 620 nm, and 650 nm, respectively.

### Determination of chlorophyll and carotenoid content

The determination of chlorophyll and carotenoid contents was performed with acetone-ethanol (1:1, V/V) mixture. About 10 mL of extracting solution was added to 0.5 g of sample, which was then placed at room temperature for 24 h in the dark, and shaken every 1 h until the color turned to white. After centrifugation at 8000 rpm and 25°C for 5 min, the absorbance was measured at the wavelengths of 470 nm, 645 nm and 663 nm, respectively, and the content of each pigment was calculated according to the corrected formula.

### Determination of total flavonoid content

Determination of total flavonoid content in peach flesh was performed according to the method described previously with minor modification as follows (Zhao et al., 2009). About 0.2 g of sample was weighed, followed by the addition of 2 mL extracting solution (70% methanol: 2% Formic acid, v/v), ground and extracted in an ice bath. The obtained sample was transferred to a centrifuge tube, added with 3 mL of extracting solution to rinse the mortar, and subjected to ultrasonic extraction for 30 min. Then, the sample was shaken at 250 rpm for 2 h at 30°C, centrifuged at 8000 rpm for 10 min, and the supernatant was filtered with a 0.45 μm filter, and stored at 4°C until use. Next, 200 μl of extract, 1.3 mL of methanol, 100 μL of NaNO_3_ (0.5M), and 100 μL of AlCl_3_ (0.3M) were added into the centrifuge tube and mixed thoroughly. After 5 min, 500 μL of NaOH (1 M) was added, and the absorbance was measured at 510 nm using rutin as the standard. The results were expressed as Rutin Equivalents (RE).

### Carotenoid-targeted metabolomic analysis

About 50 mg of sample was weighed into a 1.5-mL centrifuge tube, followed by the addition of 10 μL [13C10]-β-carotene internal standard working solution at a concentration of 20 μg/mL. Then, the extract (n-hexane: acetone: ethanol, 1: 1: 1, v/v/v, with 0.01% BHT) was added to the centrifuge tube and mixed thoroughly with a vortexer for 20 min at room temperature. After that, the sample was centrifuged at 12,000 r/min for 5 min at 4°C, and the supernatant was removed after centrifugation. The supernatant was reconstituted with 100 μL of methanol-methyl tert-butyl ether mixture (1:1, v/v). After reconstitution, it was filtered through a 0.22 μm filter, and stored in a brown injection bottle for subsequent HPLC-MS/MS analysis. Analysis of sample extracts was carried out using the UPLC-APCI-MS/MS system (SCIEX, www.sciex.com.cn). The conditions for chromatographic analysis were as follows. The chromatographic column was YMC C30 (3 μm, 100 mm × 2.0 mm i.d.); the column temperature was 28°C; the flow rate was 0.8 mL/min; the injection volume was 2 μm. Mobile phase A was methanol and acetonitrile (1: 3, v/v) with 0.01% BHT and 0.1% formic acid; Mobile phase B was methyl tert-butyl ether (with 0.01% BHT). The gradient elution program was set as: 0–3 min, 100% A; 5 min, 30% A, 70% B; 9 min, 5% A, 95% B; 10–11 min, 100% A. The mass spectrometry conditions were as follows: atmospheric pressure chemical ionization source (APCI) temperature 350°C, and curtain gas (CUR) 25 psi. In the Q-Trap 6500+, each transition was scanned based on optimized de-clustering potential (DP) and collision energy (CE).

### Correlation analysis between carotenoids and other pigments with color parameters

The L* (brightness), a* (red and green), b* (yellow and blue), c* (color saturation) and h° (hue angle) of peach pulp were measured by NH-200 colorimeter (Sine Image). Five fruit were measured in each period with three replicates. A higher L* value indicates a higher brightness. A higher a* value represents darker red, and a lower a* value stands for darker green. A higher b* value represents darker yellow, and a lower b* value stands for darker blue. A higher value of c* indicates higher color saturation. h° value varied from 0° to 180°, with 0° representing fuchsia, 90° representing yellow, and 180° representing green.

### RNA extraction, library construction, and sequencing

Total RNA extraction from samples was performed using the Trizol extraction method. The degradation of RNA was determined and DNA contamination was analyzed by 1% agarose-gel electrophoresis. RNA concentration was determined using the Qubit2.0 Fluorometer and RNA integrity was assessed using the RNA Nano 6000 Assay Kit of the Bioanalyzer 2100 system (Agilent Technologies, CA, USA). A total of 1 μg RNA per sample was used as input for the RNA sample preparations. Sequencing libraries were generated using NEBNext^®^ UltraTM RNA Library Prep Kit for Illumina^®^ (NEB, USA) following the manufacturer’s recommendations, and index codes were added to attribute sequences to each sample. The clustering of the index-coded samples was performed on a cBot Cluster Generation System using TruSeq PE Cluster Kit v3-cBot-HS (Illumia) according to the manufacturer’s instructions. After cluster generation, the libraries were sequenced on an Illumina Hiseq platform and 125 bp/150 bp paired-end reads were generated.

### Sequencing data analysis

The raw data were filtered with fastp v0.19.3 to filter out adapter-linked and low-quality reads. Paired reads with N content >10% were removed. When the number of low-quality (Q ≤ 20) bases in a read was > 50%, the paired reads were removed. All subsequent analyses were performed on the basis of clean reads. The peach reference genome and annotation files were downloaded from the GDR website (https://www.rosaceae.org/). After indexing, clean reads were compared with the reference genome. Gene alignments were calculated using feature Counts v1.6.2, and the FPKM for each gene was calculated. Samples were analyzed for differential expression using DESeq2 v1.22.1, and the P values were corrected using the Benjamini & Hochberg method. P-values and |log^2^ fold change| were used as criteria to screen for significant differential expression. Based on the hypergeometric test method, the hypergeometric distribution test was performed for KEGG in the unit of pathway.

### qRT-PCR verification

The peach reference genome was also used to establish a local database. Sequence probes were designed based on the reported genes related to carotenoid pathway, and BLASTP was compared with the local database to obtain the best matching results of genes related to carotenoid metabolism pathway. To verify the reliability of the sequencing data, 14 genes related to the carotenoid metabolic pathway were selected for quantitative reverse transcription-polymerase chain reaction (qRT-PCR) analysis performed in CFX96 real-time fluorescent quantitative PCR system (Bio-Rad, https://www.bio-rad.com/). The operation steps refer to the SYBR^®^ Premix Ex Taq manual, and the annealing temperature was 60°C. The relative expression levels of the genes were calculated using the 2^-△△Ct^ method (Schmittgen and Livak, 2008). The sequences of oligonucleotide primers used in this study are presented in **Supplementary Table S1**.

## Results

### Carotenoid content causes different flesh colors in hybrid F_1_ generation

**Figure 1:**
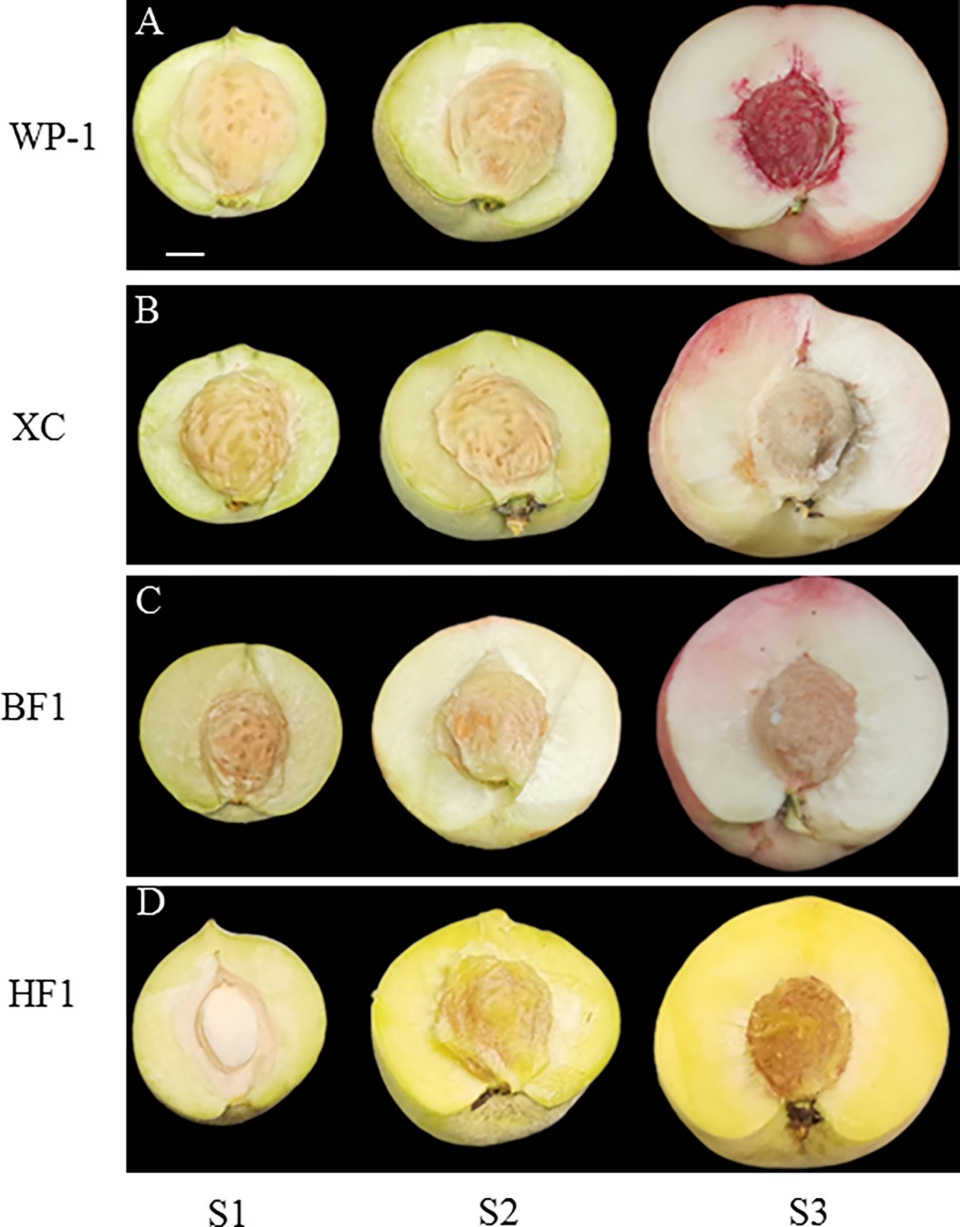
Phenotypes of Chang Song White Peach, ‘Xia Cui’ and their hybrid F1 generations at different fruit development stages. **(A)** Chang Song White Peach (WP-1). **(B)** ‘Xia Cui’ (XC). **(C)** White-fleshed peachs F1 generation (BF1). **(D)** Yellow-fleshed peachs F1 generation (HF1). S1 presents core-hardening stage. S2 stands for color-transformation stage. S3 representing mature. Bars = 1cm.

The flesh color of both WP-1 (female parent) and XC (male parent) was white. However, the flesh color was segregated into white (BF1) and yellow (HF1) in the hybrid F_1_ generation (**Figure 1**). To further elucidate the reasons for the segregation of flesh color, we determined the contents of chlorophyll, anthocyanin, flavonoid and carotenoid in the flesh of parents and hybrid F_1_ generation.

**Figure 2:**
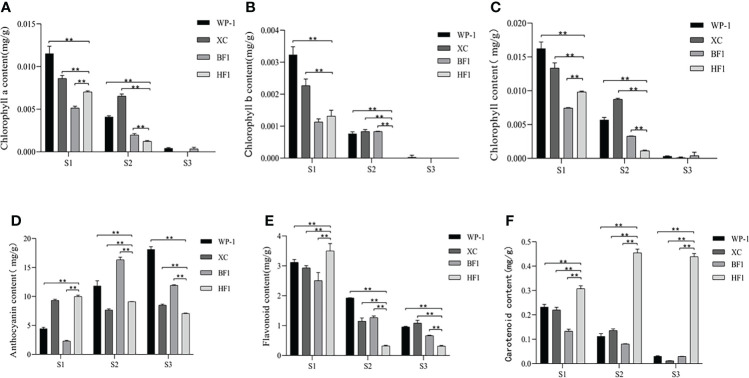
Changes of the pigment content in the flesh of the parental and hybrid F1 generations at different fruit development stages. **(A)** Chlorophyll a content. **(B)** Chlorophyll b content. **(C)** Chlorophyll content. **(D)** Anthocyanin content. **(E)** Total flavonoids content. **(F)** Carotenoid content. Data were presented as the means±standard error of three biological replicates, *means significant correlation (p < 0.05), ** means greatly significant correlation (p < 0.01).

During the three stages of fruit growth, namely the core-hardening stage (S1), color-transformation stage (S2) and mature stage (S3), the contents of chlorophyll a (Chl a), chlorophyll b (Chl b) and total chlorophyll in parental, BF1 and HF1 fruits exhibited a decreasing trend with fruit ripening (**Figures 2A–C**). The changing trend of Chl content was inconsistent with that of flesh color, particularly at S3, when there was no significant difference in Chl content between BF1 and HF1. The variation trend of anthocyanin content differed among all four materials. The anthocyanin content of the female parent WP-1 showed an increasing trend with fruit growth, while that of the male parent XC showed a first decreasing and then increasing trend. The anthocyanin content showed a first increasing and then decreasing trend in BF1, while a consistently decreasing trend in HF1 (**Figure 2D**). During fruit growth, the content of total flavonoids decreased in both the parental and F_1_ generation fruits, and the changing trend was the most obvious in HF1 (**Figure 2E**).

Along with fruit ripening, the carotenoid content gradually decreased in the parental and BF1 fruits, while gradually increased in HF1 fruit. At the S3 stage, the carotenoid content was extremely low in the parental and BF1 fruit, while still high in HF1 fruit without degradation (**Figure 2F**). These results suggested that carotenoids may be the primary reason for the different fruit flesh colors of the hybrid F_1_ generation.

### Detection and principal component analysis of carotenoids in hybrid F_1_ generation

**Figure 3:**
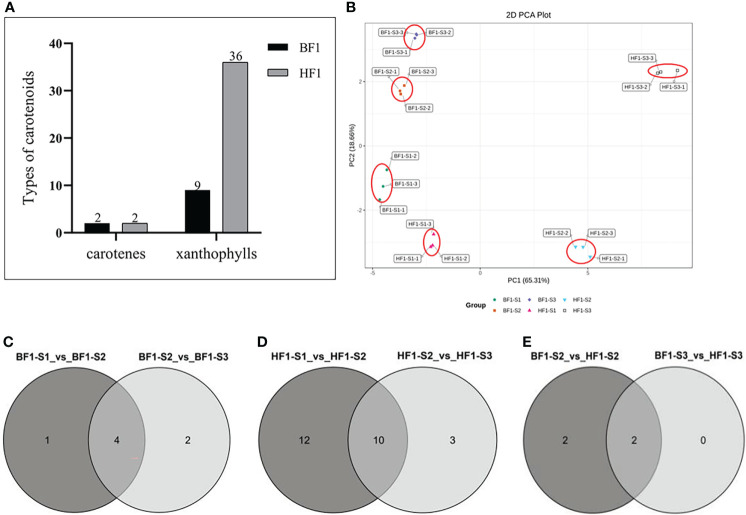
Targeted carotenoid metabolomic analysis in Hybrid F1 generation. **(A)** Detection of carotenoid species in F1 generation of peach hybrid. **(B)** Principal component analysis (PCA) biplot showing carotenoid clusters of the furit developmental stages of the peach hybrid F1 generation. **(C)** Venn diagram of differential carotenoid metabolites in the BF1 fruit development stages. **(D)** Venn diagram of differential carotenoid metabolites in the HF1 fruit development stages. **(E)** Venn diagram of differential carotenoid metabolites in fruit development stages of BF1 vs HF1.

HPLC-MS/MS (ultra-high-performance liquid chromatography-tandem mass spectrometry hyphenated with high resolution mass spectrometer) platform-targeted metabolomic technology was used to identify carotenoids in the flesh of hybrid F_1_ generation. A total of 39 carotenoid metabolites were detected, including two kinds of carotene and 37 kinds of lutein (**Table S2**). The content of these metabolites in the hybrid F_1_ generation with different flesh colors were different. The same types of carotene were detected in BF1 and HF1, namely β-carotene and phytoene. HF1 contained 36 kinds of lutein, while BF1 only had nine kinds of lutein (**Figure 3A**). BF1 and HF1 had different carotenoid metabolic profiles, which might be related to the flesh color.

Principal component analysis (PCA) could preliminarily reveal the overall metabolic differences and variability of carotenoids in BF1 and HF1. The PCA provided visualization of these metabolite data of BF1 and HF1 with the first two components PC1 + PC2 (65.31 and 18.66% of the variance, respectively). PC1 displayed that HF1 had obvious separation at different fruit development stages, while BF1 showed no obvious separation between different development stages. BF1 and HF1 were significantly segregated at the same fruit development stage. PC2 revealed that BF1 had obvious segregation at different fruit development stages, while HF1 was only segregated at S3 (**Figure 3B**).

### Heat map cluster analysis of carotenoids in hybrid F_1_ generation

**Figure 4:**
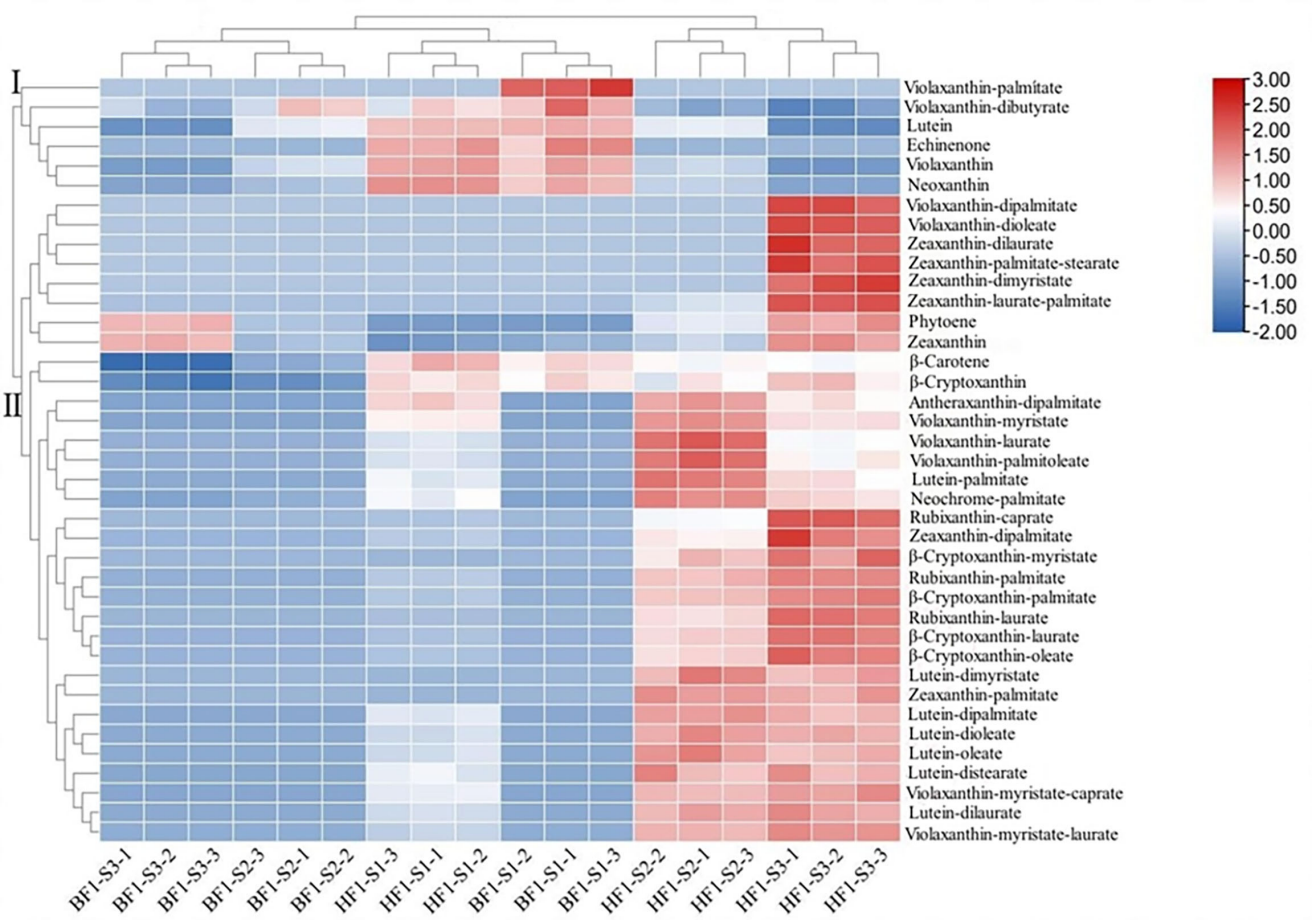
Clustering heat map analysis of carotenoid metabolites in hybrid F1 generation peach fruit.

The differences in carotenoid metabolites in the flesh of BF1 and HF1 were analyzed by clustering heat map (**Figure 4**). The carotenoid metabolites were divided into two main clusters (cluster I and cluster II) and five sub-clusters. In cluster I, violaxanthin and its derivatives (violaxanthin-palmitate, violaxanthin-dibutyrate), lutein, echinenone and neoxanthin were the main carotenoids. Cluster II mainly comprised violaxanthin derivatives (violaxanthin dipalmitate, violaxanthin dioleate, violaxanthin myristate, violaxanthin laurate, violaxanthin palmitoleate, violaxanthin-myristate-caprate, violaxanthin-myristate-laurate), zeaxanthin and its derivatives (zeaxanthin dilaurate, zeaxanthin-palmitate-stearate, zeaxanthin dimyristate, zeaxanthin-laurate-palmitate, zeaxanthin dipalmitate, zeaxanthin palmitate), β-carotene, phytoene, β-cryptoxanthin and its derivatives (β-cryptoxanthin myristate, β-cryptoxanthin palmitate, β-cryptoxanthin laurate, β-cryptoxanthin oleate), antheraxanthin dipalmitate, lutein derivatives (lutein palmitate, lutein dimyristate, lutein dipalmitate, lutein dioleate, lutein oleate, lutein distearate, lutein dilaurate), neochrome palmitate, and rubixanthin derivatives (rubixanthin caprate, rubixanthin palmitate, rubixanthin laurate). Carotenoid metabolites began to show differences in flesh color between BF1 and HF1 at S2 stage. HF1 had significantly higher contents of carotenoid metabolites than BF1, as well as many unique metabolites. BF1 had the highest content and most types of carotenoids at the S1 stage, while the carotenoid content and types gradually decreased at the S2 and S3 stages. Besides, the content and types of carotenoids continuously increased in HF1, and became the most abundant at the S3 stage. These results were consistent with the previously detected changes in the total amount of carotenoids.

### Analysis of differential carotenoid metabolites

The differential carotenoid metabolites were screened by using Fold_Change. A metabolite with fold change ≥ 2 or ≤ 0.5 in the two groups was identified as a differential metabolite. In BF1, a total of five differential metabolites were significantly down-regulated in S2 vs. S1. There were six differential metabolites in S3 vs. S2, among which two were significantly up-regulated and four were significantly down-regulated (**Table S3**). There were four common differential metabolites in the three fruit development stages (**Figure 3C**), including β-carotene, violaxanthin, neoxanthin and lutein, which gradually decreased with the growth and development of fruit in BF1 (**Table S4**). In HF1, there were 22 differential metabolites, among which 19 were significantly up-regulated and three were significantly down-regulated in S2 vs. S1. In S3 vs. S2, a total of 13 differential metabolites were found, of which seven were significantly up-regulated and six were significantly down-regulated. There were ten common differential metabolites in the three fruit development stages (**Figure 3D**), including ruthexanthin caprate, rubixanthin laurate, violexanthin laurate, violexanthin myristate, violexanthin palmitoleate, zeaxanthin dipalmitate, β-cryptoxanthin oleate, violexanthin, neoxanthin and lutein. Among them, rubixanthin caprate, rubixanthin laurate, zeaxanthin dipalmitate and β-cryptoxanthin oleate gradually increased with fruit development, and therefore could be considered as key metabolites affecting carotenoid accumulation in HF1 (**Table 1**).

**Table 1:** Changes of differential carotenoid metabolite content in HF1 at different fruit development stages (µg/g).

Metabolite	HF1-S1	HF1-S2	HF1-S3	Fold_Change	
				SI_vs_S2	S2_vs_S3
Rubixanthin caprate	0.02±0.00	0.13±0.00	0.42±0.02	5.81	3.18
Rubixanthin laurate	0.07±0.00	0.89±0.05	2.07±0.12	13.31	2.33
Violaxanthin laurate	0.14±0.01	0.62±0.03	0.21±0.01	4.34	0.34
Violaxanthin myristate	2.85±0.13	8.12±0.45	3.49±0.11	2.85	0.43
Violaxanthin palmitoleate	0.05±0.00	0.18±0.01	0.08±0.10	3.88	0.44
Zeaxanthin dipalmitate	0.03±0.00	0.12±0.01	0.27±0.05	4.05	2.23
β-Cryptoxanthin oleate	0.11±0.01	0.96±0.07	2.04±0.22	8.77	2.13
Violaxanthin	1.25±0.05	0.41±0.03	0.08±0.01	0.33	0.22
Neoxanthin	1.68±0.02	0.32±0.02	0.02±0.00	0.19	0.07
Lutein	23.28±1.01	7.15±0.25	0.60±0.03	0.31	0.08

At different fruit development stages, the number of differential metabolites between BF1 and HF1 is presented in **Figure 3E**. No differential metabolite was found between BF1 and HF1 at the S1 stage. β-carotene and β-cryptoxanthin were two common differential metabolites at S2 and S3, and they were both significantly lower in BF1 than in HF1 (**Table S5**).

### Screening of core carotenoid metabolites based on correlation analysis of phenotype, content and changing trend

**Figure 5:**
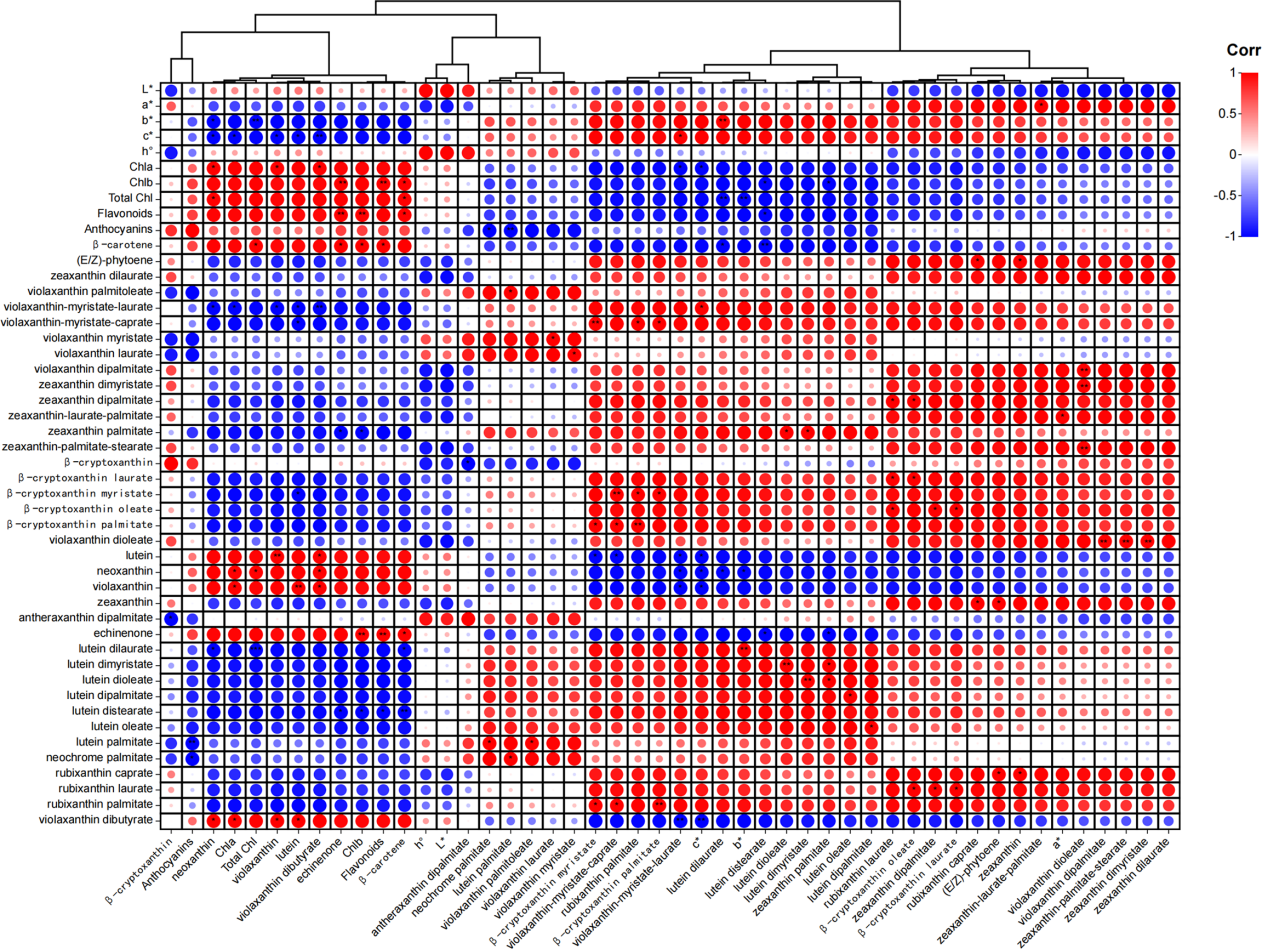
Correlation analysis between color difference parameters, carotenoid components and other pigments in HF1. The higher the value of L, the higher the brightness. The larger the value of a*, the darker the red, the smaller the value of a*, the darker the green. The larger the value of b*, the darker the yellow, the smaller the value of b*, the darker the blue. The higher the value of c*, the higher the color saturation. h° values vary from 0° to 180°, with 0° representing fuchsia, 90° representing yellow, and 180° representing green. *means significant correlation (p < 0.05), ** means greatly significant correlation (p < 0.01).

Different pigments have different contributions to pulp color. To screen out the core carotenoid metabolites leading to the color change in yellow-fleshed peach, we conducted a correlation analysis between color difference parameters, carotenoid components and other pigments in HF1 (**Figure 5**) and BF1 (**Figure S1**). The b* represents the yellow-blue difference index, with a higher value indicating a darker yellow and a lower value indicating a darker blue.

The results showed that most carotenoid components (31 out of 38), C* (color saturation) and total carotenoid content were positively correlated with b* in HF1. Among the 31 carotene components, the top 10 components with the highest correlation with b* included lutein dilaurate, lutein distearate, violaxanthin-myristate-laurate, zeaxanthin palmitate, violaxanthin-myristate-caprate, β-cryptoxanthin myristate, lutein dimyristate, lutein dioleate, rubixanthin palmitate and β-cryptoxanthin palmitate. However, the contents of β-carotene, lutein, flavonoids, chlorophyll a and violaxanthin were significantly positively correlated with b* in BF1. These results help a better understanding of the color formation of peach flesh with different carotenoid components.

An analysis of differential metabolites resulted in the screening of four carotenoid components as the main substances for the increase in carotenoids in HF1 pulp. The correlation coefficients of rubixanthin caprate, rubixanthin laurate, zeaxanthin dipalmitate and β-cryptoxanthin oleate with b* were 0.79, 0.87, 0.85 and 0.89, respectively. Besides, the correlation coefficients of β-carotene and β-cryptoxanthin with b* in BF1 pulp were 0.99 and 0.98, respectively. The correlation analysis results again demonstrated that these carotenoid components are closely related to the formation of flesh color traits in F_1_ hybrids.

### Transcriptome sequencing data assembly and annotation

Transcriptome sequencing analysis of fruit samples was carried out on hybrid F_1_ generation. A total of 1179518038 raw reads were obtained from the sequencing of all samples, and a total of 1127149218 clean reads were filtered. The alignment rate of each sample was greater than 97%, and the overall sequencing error rate was as low as 0.03%. The percentage of Q20 bases in each sample was over 97%; the percentage of Q30 bases was above 92%; and the GC content accounted for 45.85%–46.48% (**Table S6**). The assembled transcripts were annotated in the KEGG, Swiss-Prot, NR, KOG, and GO databases, resulting in 19561, 19572, 27553, 25535, and 22092 annotated transcripts, respectively. A total of 16,657 transcripts were co-annotated across all databases (**Figure S2**). PCA analysis showed that the biological replicates of different samples were clustered together at the same fruit development stage with high reproducibility. At different developmental stages, BF1 and HF1 were clearly separated, indicating that the transcripts for different flesh colors have obvious specificity (**Figure S3A**). Furthermore, the biological reproducibility was evaluated using Pearson’s correlation coefficient. R^2^ was approximately 1 between repetitions of the same sample, indicating that the sample had strong repeatability and correlation. However, there were weak correlations between different samples, which can reflect the differences between samples (**Figure S3B**). Taken together, the transcriptome sequencing data obtained in this study were of high quality and reliability.

### Identification of differentially expressed genes in carotenoid metabolic pathways

Differentially expressed gene (DEG) analysis was performed on the transcriptome data to identify the genes associated with carotenoid metabolic pathways (**Table S7**). Relative to those in BF1, 426 genes were significantly up-regulated, and 217 genes were significantly down-regulated in HF1 at the S1 stage. Among these DEGs, a total of five DEGs were related to carotenoid metabolism pathway, including *NCED* (Prupe.1G255500_v2.0.a1), *CCD8* (Prupe.1G448400_v2.0.a1), *ABA2* -1 (Prupe.6G138700_v2.0.a1), *ABA2-2* (Prupe.6G286600_v2.0.a1) and *ZEP* (Prupe.6G162100_v2.0.a1). The expression levels of *NCED* and *CCD8* were significantly down-regulated, and those of *ABA2-1, ABA2-2* and *ZEP* were significantly up-regulated in HF1 (**Table S8**). Relative to those in BF1, 191 and 237 genes were significantly up-regulated and down-regulated in HF1 at the S2 stage, respectively. Among them, two DEGs were associated with carotenoid metabolism pathway, including *NECD* and *CYP707A* (Prupe.5G013100_v2.0.a1). The expression levels of both genes were significantly decreased in HF1 (**Table S9**). Relative to those in BF1, 209 and 546 genes were significantly up-regulated and down-regulated in HF1 at the S3 stage, respectively. Similar to the S2 stage, the DEGs related to the carotenoid metabolic pathway were also *NECD* and *CYP707A*. The expression of *NECD* and *CYP707A* was continuously significantly down-regulated in HF1, and that of *NCED* was down-regulated by 3.7 folds (**Table S10**).

### Analysis of genes related to carotenoid metabolic pathway

**Figure 6:**
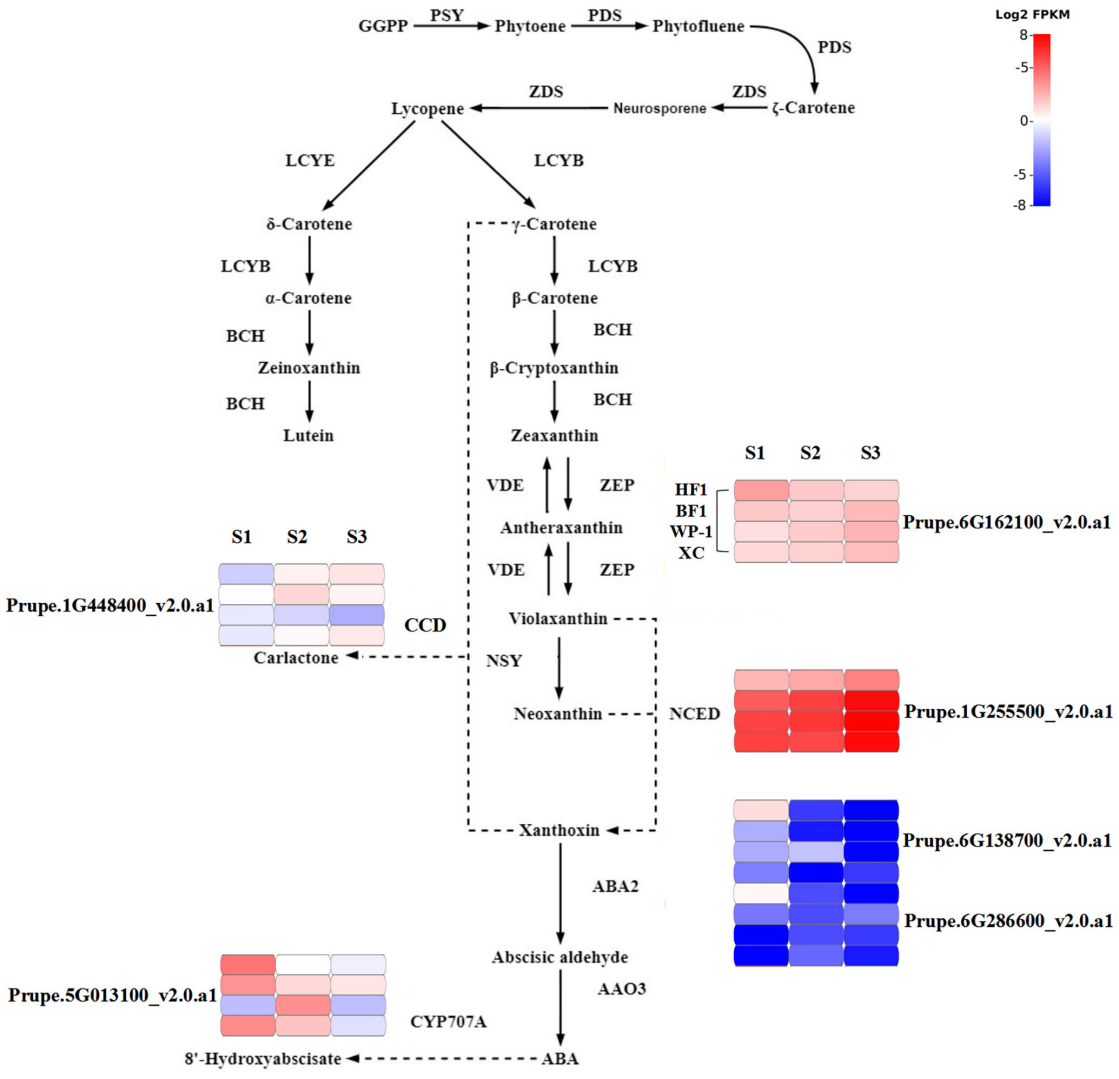
Changes in gene expression levels mapped to carotenoid metabolism was shown of BF1 and HF1 at fruit development stages.

In the metabolic pathway of carotenoids, we determined the expression levels of the aforementioned DEGs at three fruit development stages of parents and hybrid F_1_ generation (**Figure 6**). In BF1, the expression of *ZEP* first increased and then decreased; while that of *CCD8* showed a first decreasing and then increasing trend; *ABA2* and *CYP707A* showed continuous decreases in expression; and only the expression of *NCED* continuously increased. In HF1, the expression of *ZEP*, two *ABA2s* and *CYP707A* showed a decreasing trend, while that of *CCD8* and *NCED* exhibited an increasing trend. In both BF1 and HF1, changes in the expression of *ZEP* and *CCD8* were not completely consistent with those in carotenoid content.

The expression levels of *ABA2* and *CYP707A* were low but different between HF1 and BF1. S2 and S3 were two stages with the greatest difference in flesh color between HF1 and BF1. However, enzyme-coding genes (*ABA2-1*, *ABA2-2* and *CYP707A*) with low expression levels may not be involved in regulating the flesh color. Compared with that in BF1 and two parents, *NCED* was significantly down-regulated during the development of HF1, and the fold change continuously became more significant at the three stages along with flesh development. The changes in *NCED* expression in BF1 and HF1 were consistent with those in flesh color. Hence, the *NCED* gene may be involved in regulating flesh color in the F_1_ generation of peach hybrid.

### Integrated correlation analysis of differentially expressed genes and core differential metabolites

**Figure 7:**
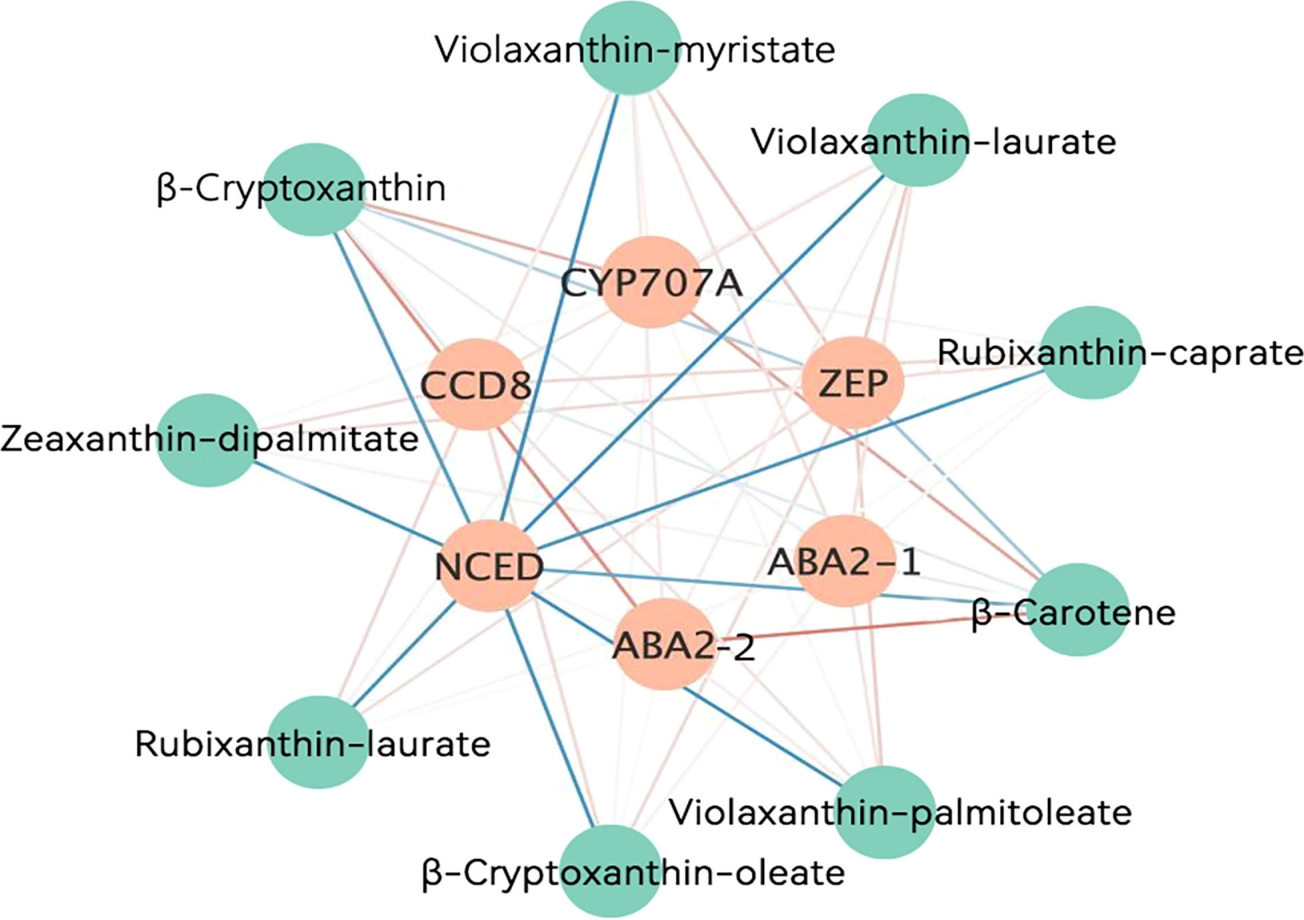
Correlation analysis between DEGs and core differential carotenoid metabolites. The blue lines represent negative correlations. The yellow lines represent positive correlations. The darker the color, the higher the correlation coefficient.

An integrated correlation analysis of DEGs with six core metabolites and three main differential violaxanthin derivatives in HF1 was performed to understand the relationship between the DEGs and differential carotenoid metabolites (**Figure 7**). As a result, the expression levels of *NCED* at the three stages of flesh development were significantly negatively correlated with the contents of all core carotenoid metabolites. The expression level of *NCED* was significantly down-regulated in HF1, indicating that it may negatively regulate the accumulation of carotenoids in peach flesh, thereby contributing to the formation of yellow flesh. In addition, *CCD8*, *ZEP*, two *ABA2s*, and *CYP707A* were weakly correlated with core differential metabolites, indicating that they were not the main regulatory genes for carotenoid accumulation.

### qRT-PCR validation of transcriptomic data

**Figure 8:**
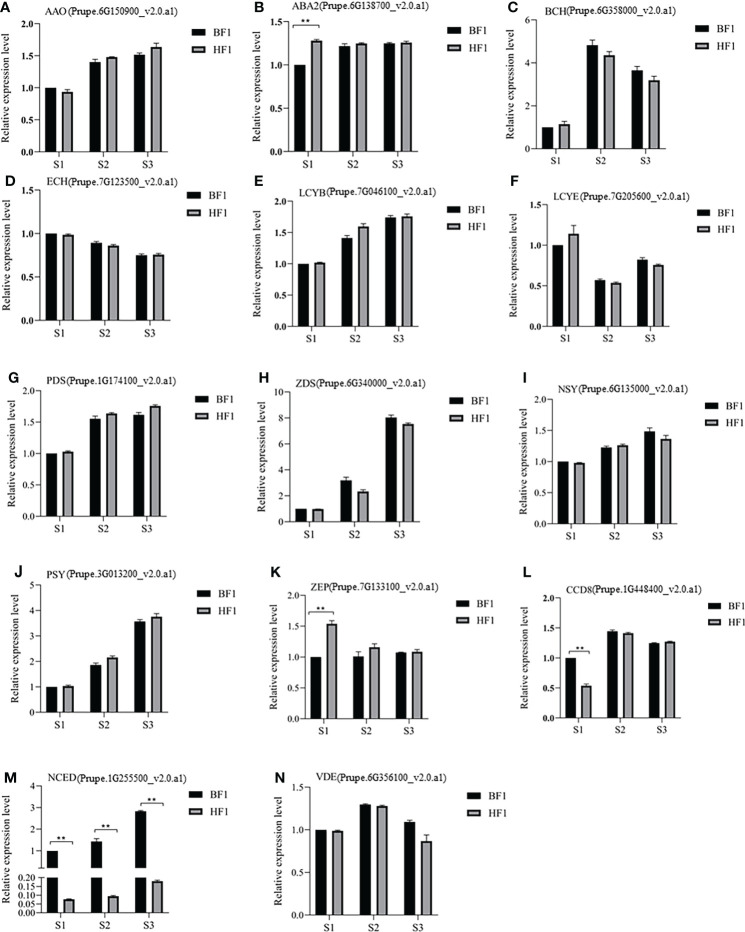
qRT-PCR analysis of the expression of genes related with carotenoids metabolism in BF1 and HF1 at fruit development stages. **(A)** AAO. **(B)** ABA2. **(C)** BCH. **(D)** ECH. **(E)** LCYB. **(F)** LCYE. **(G)** PDS. **(H)** ZDS. **(I)** NSY. **(J)** PSY. **(K)** ZEP. **(L)** CCD8. **(M)** NCED. **(N)** VDE. Data were presented as the means±standard error of three biological replicates, *means significant difference at p < 0.05 by t-test; ** means significant difference at p < 0.01 by t-test.

qRT-PCR validation of 14 genes in the carotenoid metabolic pathway was carried out (**Figure 8**). *ABA2*, *NCED*, *ZEP* and *CCD8* had significantly different expression levels between BF1 and HF1, and the remaining genes showed no difference. There were significant differences in the relative expression levels of *ABA2*, *ZEP* and *CCD8* at the S1 stage. However, *NCED* showed significant differences throughout the fruit development. These results were generally consistent with the transcriptome data results, indicating that the transcriptome data were authentic and credible. Therefore, the transcriptome data obtained in this study can be used to study carotenoid metabolism-related genes in F_1_ hybrid peach.

## Discussion

Carotenoid content greatly determines the flesh color of peach (Gil et al., 2002; Giberti et al., 2019; Sun and Li, 2020; Qin et al., 2021). The carotenoid content in yellow-fleshed peach is significantly higher than that in white-fleshed peach (Vizzotto et al., 2006). With the development of fruit, the carotenoid content gradually increased and reaches the highest at the ripening stage of yellow-fleshed peach, while gradually decreases to the lowest at the ripening stage of white-fleshed peach. In this study, carotenoids were the main pigments in yellow-fleshed peach fruit (HF1), which increased with fruit development, and their content was significantly higher than that in the parent and white-fleshed F_1_ generation (BF1). In the parents and BF1, the content of carotenoids decreased with fruit development. Furthermore, the contents of chlorophyll, anthocyanins and total flavonoids were not consistent with the changes in flesh color, and thus not the main pigments affecting the segregation of flesh color in F_1_ hybrid generation. In general, carotenoid is the main pigment that affects the color of the flesh of peach hybrids, which is consistent with the results in previous studies (Brandi et al., 2011).

The contents of lutein, zeaxanthin, β-carotene and β-cryptoxanthin gradually increased with the fruit development of yellow-fleshed peach, while rather low carotenoid levels were detected in white-fleshed peach (Cao et al., 2017). The accumulation of antheraxanthin and violaxanthin in yellow-fleshed peach was significantly higher than that in white-fleshed peach, and the difference in the content of these two carotenoids may be the main reason for the different colors of the flesh (Ma et al., 2014). In this study, metabolomics analysis revealed that the contents of β-carotene, β-cryptoxanthin, β-cryptoxanthin oleate, rubixanthin caprate, rubixanthin laurate and zeaxanthin dipalmitate in HF1 were significantly higher than those in BF1 after the color-transformation stage. Among them, the core carotenoid components leading to the yellow flesh color of HF1 include rubixanthin caprate, rubixanthin laurate, zeaxanthin dipalmitate and β-cryptoxanthin oleate. The core substances leading to the decrease in carotenoid content in BF1 are β-carotene and β-cryptoxanthin. Moreover, our results and previous research results together indicate that there are diverse accumulation patterns of carotenoids in different peach varieties.

In this study, the DEGs related to carotenoid metabolism in BF1 and HF1 were identified by transcriptome analysis. Among the DEGs, *CYP707A* and two *ABA2s* are involved in the decomposition of carotenoids to abscisic acid, and therefore more closely related to the synthesis of abscisic acid. Given their extremely low expression levels after color-transformation stage to maturity, the decomposition of carotenoids caused by *CYP707A* and the two *ABA2s* may be limited.

*CCD* family genes have been found to affect fruit flesh color through degradation of carotenoids. *CCD4* is a gene controlling peach flesh color, and its expression in the white-fleshed peach leads to the degradation of carotenoids, while it is inactive in yellow-fleshed peach (Ma et al., 2014). In addition, identification and analysis of the *CCD4* gene in white-fleshed peach and its yellow-fleshed mutant have demonstrated that the *CCD4* gene was mutated and became inactive in yellow-fleshed peach, which blocked the decomposition pathway of carotenoids, resulting in a yellow flesh color (Adami et al., 2013). Wen et al. revealed the differences in the expression patterns of the *CCD4* gene between the white-fleshed ‘Piqiutao’ and its yellow-fleshed mutant. The yellow-fleshed peach mutant had a significantly lower expression level of *CCD4* than white-fleshed ‘Piqiutao’, resulting in its yellow flesh phenotype (Wen et al., 2020). In this study, the transcriptome analysis of BF1 and HF1 revealed that *CCD8* is probably the gene in the *CCD* family that affects the flesh color of the F_1_ hybrid generation. Previous studies have suggested that CCD8 may use β-carotene as a substrate to catalyze the synthesis of strigolactone, thereby regulating cell division and controlling shoot differentiation (Mason, 2013; Ren et al., 2020). In the carotenoid metabolic pathway, γ-carotene can be catalyzed by hydroxylase to generate ruthexanthin (Brown and Weedon, 1968), and can also be catalyzed by LCYB to generate β-carotene (Koltai and Kapulnik, 2011; Moreno et al., 2013), which can be further catalytically utilized by CCD8. In this study, the *CCD8* gene was significantly down-regulated at the S1 stage in HF1 compared with that in BF1, which inhibited the decomposition of carotenoids. However, *CCD8* showed no significant difference in expression between HF1 and BF1 at the S2 and S3 stages, and even had a significantly higher expression level in HF1 than in the white-fleshed parent (WP-1). Therefore, *CCD8* is not the key gene responsible for the difference in flesh color between BF1 and HF1 in this study.

ZEP is an enzyme in the carotenoid metabolic pathway that catalyzes zeaxanthin to antheraxanthin and then to violaxanthin (Kato et al., 2004). Inhibition or silencing of *ZEP* expression could increase the carotenoid content in fruit (Römer et al., 2002; Lee et al., 2021). In this study, compared with the white-flesh F_1_ generation, *ZEP* was significantly up-regulated in HF1 only in the S1 stage, and the content of violaxanthin in HF1 was significantly higher than that of BF1 at this stage. This study also suggested that the up-regulated expression of *ZEP* can promote the synthesis of violaxanthin, thereby promoting the accumulation of carotenoids. However, the expression of *ZEP* was not significantly different between BF1 and HF1 at the S2 and S3 stages, which is inconsistent with the changes in carotenoid content. Hence, *ZEP* is also not the key gene responsible for the difference in flesh color between BF1 and HF1.

NCED plays a role in decomposing carotenoids and providing precursors for the synthesis of ABA. NCED negatively regulates the content of carotenoids, and may affect the ripening of fruit by regulating the synthesis of ABA (Zhang et al., 2009; Ji et al., 2014). Down-regulation of *NCED* resulted in carotenoid accumulation in tepals of Chinese narcissus mutants (Zhang et al., 2022). So far, there have been few reports about the regulation of NCED on fruit flesh color. Jiang et al. for the first time revealed that the *NCED* gene in the extended pathway of β-carotenoid metabolism is a key gene regulating the color formation of apricot flesh (Jiang et al., 2019). In the present study, only *NCED* expression was significantly down-regulated throughout the development in HF1 compared with that in BF1. Moreover, the carotenoid content of HF1 was significantly higher than that of BF1 during the whole fruit development. In addition, the fruit growth period of HF1 was delayed by 5 d compared with that of BF1, which is similar to the effect of down-regulated expression of *NCED* on fruit phenotype in other species. In HF1, down-regulation of *NCED* inhibited the conversion of violaxanthin to neoxanthin, resulting in an increase in the total carotenoid content. Down-regulated expression of *NCED* also decreases the content of precursors for ABA synthesis, resulting in insufficient ABA and prolonging the ripening period of HF1. Therefore, the down-regulation of *NCED* gene leads to the accumulation of carotenoids in HF1 to form a yellow flesh trait, and *NCED* is a key gene regulating yellow flesh trait.

## Conclusion

In this study, we used the peach F_1_ hybrid population with segregation of flesh color and their parents to conduct carotenoid-targeted metabolomics analysis and transcriptome sequencing at three key stages of flesh color conversion. Four core carotenoid components (rubixanthin caprate, rubixanthin laurate, zeaxanthin dipalmitate and β-cryptoxanthin oleate) leading to yellow flesh and two core components (β-carotene and β-cryptoxanthin) responsible for the decrease in carotenoid content in BF1 were screened by correlation analysis of phenotype, carotenoid content and changing trend. Joint transcriptome and metabolome analysis revealed that the high expression of *ZEP* at the S1 stage of HF1 leads to a higher content of violaxanthin than that in BF1, which contributes to the accumulation of carotenoid metabolic intermediates in HF1 flesh. Moreover, *NCED* was significantly down-regulated during the entire development stage of HF1, inhibiting the decomposition of carotenoid metabolic intermediates and resulting in a substantial increase in the content of violaxanthin and its upstream carotenoid metabolites, finally contributing to the formation of yellow-fleshed trait.

## Data availability statement

The RNA-seq data presented in the study are deposited in the NGDC repository (https://ngdc.cncb.ac.cn/gsa), accession numbers CRA008431 and CRA008459.

## Author contributions

HS was responsible for the metabolic and transcriptomic analyses, and manuscript preparation. GJ, SS, JingL, MT, LW and ZX performed the hybrid assays. YZ, WT, WY and HC performed the qRT-PCR and constructed discussions. JunL was responsible for measurement of pigments content. CC and ML prepared some figures. RG and DC were responsible for experiment design and reviewed the manuscript. All authors contributed to the article and approved the submitted version.

## Funding

This work was funded by National Peach Industrial Technology System (CARS-31-Z-12), Sichuan Science and Technology Support Plan (21ZDYF2196), Sichuan Youth Science and technology innovation research team (20CXTD0041).

## Conflict of interest

The authors declare that the research was conducted in the absence of any commercial or financial relationships that could be construed as a potential conflict of interest.

## Publisher’s note

All claims expressed in this article are solely those of the authors and do not necessarily represent those of their affiliated organizations, or those of the publisher, the editors and the reviewers. Any product that may be evaluated in this article, or claim that may be made by its manufacturer, is not guaranteed or endorsed by the publisher.
