# Supplementary material for: Down-regulation of NCED leads to the accumulation of carotenoids in the flesh of F1 generation of peach hybrid
Source: Front Plant Sci. 2022 Nov 3;13:1055779. doi: 10.3389/fpls.2022.1055779 (PMC9669654; doi:10.3389/fpls.2022.1055779)
Supplement: Supplementary file 1 [file DataSheet_1.docx]

**Supplementary Table 1.** Oligonucleotide primers used in this study for qRT-PCR.

| Gene | Upstream primer sequence (5' - 3') | Downstream primer sequence (5' - 3') |
| --- | --- | --- |
| *Actin-7* | CCTATGTAGGTGATGAAGCC | GATCTTCTCCATGTCATCCC |
| *AAO*(Prupe.6G150900_v2.0.a1) | CAATAGAAGGGGACTAAGGC | GTATAAGATCCTGCATGGGG |
| *ABA2*(Prupe.6G138700_v2.0.a1) | CAAGTGCTTTAGGAGGCTTA | ACGAATCCCATGAATTCCAA |
| *BCH*(Prupe.6G358000_v2.0.a1) | CCTGAGAGAAGCAGAAGATC | CTTCGTCTACCATCCTCTTG |
| *CCD8*(Prupe.1G448400_v2.0.a1) | CTATTGTGACAGAGACCGAG | TGGCATTTCCGGTACAATTA |
| *ECH*(Prupe.7G123500_v2.0.a1) | AGTTCTTGACACGCTGTATT | ATCTTGACCAGCATTGACTT |
| *LCYB*(Prupe.7G046100_v2.0.a1) | GCAGACGAAAAAGGATCTTG | AACAGTGATTTGTCCTCCTC |
| *LCYE*(Prupe.7G205600_v2.0.a) | CTTTGTCCTCAGCTGATCTC | GCTCCAGTAGGATCAGAAAG |
| *NCED*(Prupe.1G255500_v2.0.a1) | CTGTTAGGATCTCCAAAGGC | ATTGTATTGGCCTGTAAAGACG |
| *NSY*(Prupe.6G135000_v2.0.a1) | TGGAGAAATTCCCATCTTGG | AACACTGGCCTACTAACAAG |
| *PDS*(Prupe.1G174100_v2.0.a1) | CATATCCAGTCATTAGGCGG | CTGGAGTGGCGAATACATAG |
| *PSY*(Prupe.3G013200_v2.0.a1) | AGCATTACTATGGGTGGTTT | TCTGTACTGAGAGAGCAAGA |
| *VDE*(Prupe.6G356100_v2.0.a1) | CTGCCAATATGCTTGTCTG | GACACTGCGCATTCATTAAA |
| *ZDS*(Prupe.6G340000_v2.0.a1) | TTAGGTGGGGATGTAGAGAG | CTCTTGATTCCGGGTACATC |
| *ZEP*(Prupe.7G133100_v2.0.a) | TCGATGGAGAGTGGTATCTT | CTTGTAGCTGATACGAGCAT |

**Supplementary Table 2.** Detection of carotenoids targeted metabolites.

| Metabolite | BF1-S1 | BF1-S2 | BF1-S3 | HF1-S1 | HF1-S2 | HF1-S3 |
| --- | --- | --- | --- | --- | --- | --- |
| β-Carotene | 2.6422 | 0.7674 | 0.1467 | 3.3275 | 2.1328 | 2.0889 |
| Phytoene | N/A | 0.3462 | 2.1557 | N/A | 0.7983 | 2.5976 |
| Zeaxanthin dilaurate | N/A | N/A | N/A | N/A | N/A | 0.0437 |
| Violaxanthin palmitoleate | N/A | N/A | N/A | 0.0464 | 0.1803 | 0.0802 |
| Violaxanthin palmitate | 0.2746 | N/A | N/A | N/A | N/A | N/A |
| Violaxanthin-myristate-laurate | N/A | N/A | N/A | 0.1339 | 0.5423 | 0.6743 |
| Violaxanthin-myristate-caprate | N/A | N/A | N/A | 0.942 | 2.5969 | 3.4849 |
| Violaxanthin myristate | N/A | N/A | N/A | 2.8484 | 8.1182 | 3.4915 |
| Violaxanthin laurate | N/A | N/A | N/A | 0.1436 | 0.6239 | 0.2129 |
| Violaxanthin dipalmitate | N/A | N/A | N/A | N/A | N/A | 0.3155 |
| Zeaxanthin dimyristate | N/A | N/A | N/A | N/A | N/A | 0.0163 |
| Zeaxanthin dipalmitate | N/A | N/A | N/A | 0.0299 | 0.1212 | 0.27 |
| Zeaxanthin-laurate-palmitate | N/A | N/A | N/A | N/A | 0.0151 | 0.0967 |
| Zeaxanthin palmitate | N/A | N/A | N/A | N/A | 0.1298 | 0.1186 |
| Zeaxanthin-palmitate-stearate | N/A | N/A | N/A | N/A | N/A | 0.0114 |
| β-Cryptoxanthin | 0.3627 | 0.1419 | 0.1133 | 0.3755 | 0.3268 | 0.3951 |
| β-Cryptoxanthin laurate | N/A | N/A | N/A | 0.1028 | 1.0267 | 2.0413 |
| β-Cryptoxanthin myristate | N/A | N/A | N/A | N/A | 0.1268 | 0.1962 |
| β-Cryptoxanthin oleate | N/A | N/A | N/A | 0.1089 | 0.9552 | 2.0371 |
| β-Cryptoxanthin palmitate | N/A | N/A | N/A | 0.1375 | 0.9952 | 1.5751 |
| Violaxanthin dioleate | N/A | N/A | N/A | N/A | N/A | 0.1646 |
| Canthaxanthin | 26.2892 | 7.0902 | 0.7637 | 23.2753 | 7.1526 | 0.6041 |
| Neoxanthin | 1.2782 | 0.2021 | 0.0165 | 1.6752 | 0.3174 | 0.0233 |
| Violaxanthin | 1.119 | 0.4615 | 0.1156 | 1.2471 | 0.4082 | 0.0887 |
| Zeaxanthin | 0.5019 | 0.5996 | 1.5852 | 0.3709 | 0.7151 | 1.8055 |
| Antheraxanthin dipalmitate | N/A | N/A | N/A | 0.3641 | 0.486 | 0.3033 |
| Echinenone | 0.0032 | N/A | N/A | 0.0031 | N/A | N/A |
| Lutein dilaurate | N/A | N/A | N/A | 0.2744 | 0.9901 | 1.0813 |
| Lutein dimyristate | N/A | N/A | N/A | N/A | 0.2797 | 0.2396 |
| Lutein dioleate | N/A | N/A | N/A | 0.2841 | 1.1987 | 1.0661 |
| Lutein dipalmitate | N/A | N/A | N/A | 0.3529 | 1.1272 | 0.9406 |
| Lutein distearate | N/A | N/A | N/A | 0.0377 | 0.0807 | 0.0819 |
| Lutein oleate | N/A | N/A | N/A | 0.0774 | 0.267 | 0.2156 |
| Lutein palmitate | N/A | N/A | N/A | 0.0994 | 0.2934 | 0.1606 |
| Neochrome palmitate | N/A | 0.0444 | N/A | 0.3488 | 0.8934 | 0.549 |
| Rubixanthin caprate | N/A | N/A | N/A | 0.0227 | 0.132 | 0.4199 |
| Rubixanthin laurate | N/A | N/A | N/A | 0.0669 | 0.8899 | 2.0748 |
| Rubixanthin palmitate | N/A | N/A | N/A | 0.2251 | 1.5532 | 2.4401 |
| Violaxanthin dibutyrate | 0.1663 | 0.1382 | 0.0966 | 0.1356 | 0.0881 | 0.0729 |

**Supplementary Table 3.** Comparison of different carotenoid metabolites in BF1 and HF1 at different fruit development stages.

| Group name | Total | Up regulated | Down regulated |
| --- | --- | --- | --- |
| BF1-S1_vs_BF1-S2 | 5 | 0 | 5 |
| BF1-S2_vs_BF1-S3 | 6 | 2 | 4 |
| HF1-S1_vs_HF1-S2 | 22 | 19 | 3 |
| HF1-S2_vs_HF1-S3 | 13 | 7 | 6 |
| BF1-S1_vs_HF1-S1 | 0 | 0 | 0 |
| BF1-S2_vs_HF1-S2 | 4 | 4 | 0 |
| BF1-S3_vs_HF1-S3 | 2 | 2 | 0 |

**Supplementary Table 4**. Changes of differential carotenoid metabolite content in BF1 at different fruit development stages (μg/g).

| Metabolite | BF1-S1 | BF1-S2 | BF1-S3 | Fold_Change | |
| --- | --- | --- | --- | --- | --- |
|  |  |  |  | S1_vs_S2 | S2_vs_S3 |
| β-Carotene | 2.64±0.23 | 0.77±0.06 | 0.15±0.01 | 0.29 | 0.19 |
| Violaxanthin | 1.12±0.13 | 0.46±0.04 | 0.12±0.00 | 0.41 | 0.25 |
| Neoxanthin | 1.28±0.23 | 0.20±0.03 | 0.02±0.00 | 0.16 | 0.08 |
| Lutein | 26.29±1.53 | 7.09±0.52 | 0.76±0.05 | 0.27 | 0.11 |

**Supplementary Table 5**. Changes in differential metabolite content of the BF1 and HF1 at different fruit development stages (μg/g).

| Metabolite | Stage | BF1 | HF1 | Fold_Change |
| --- | --- | --- | --- | --- |
| β-Carotene | S1 | 2.64±0.23 | 3.33±0.42 | 1.26 |
|  | S2 | 0.77±0.06 | 2.13±0.15 | 2.78 |
|  | S3 | 0.15±0.01 | 2.09±0.10 | 14.25 |
| β-Cryptoxanthin | S1 | 0.36±0.03 | 0.38±0.01 | 1.04 |
|  | S2 | 0.14±0.01 | 0.33±0.04 | 2.30 |
|  | S3 | 0.11±0.02 | 0.40±0.03 | 3.49 |

**Supplementary Table 6.** Detection of carotenoids targeted metabolites.

| Sample | Raw Reads | Clean Reads | Reads mapped | Error Rate(%) | Q20(%) | Q30(%) | GC Content(%) |
| --- | --- | --- | --- | --- | --- | --- | --- |
| BF1-S1-1 | 65901416 | 63356826 | 61533815(97.12%) | 0.03 | 97.54 | 93.31 | 46.34 |
| BF1-S1-2 | 59410590 | 57904080 | 56403944(97.41%) | 0.03 | 97.45 | 93.01 | 46.24 |
| BF1-S1-3 | 55073034 | 53483836 | 52126543(97.46%) | 0.03 | 97.44 | 93.07 | 46.48 |
| BF1-S2-1 | 68770396 | 66146444 | 64465071(97.46%) | 0.03 | 97.59 | 93.4 | 46.23 |
| BF1-S2-2 | 67624546 | 60799240 | 59130535(97.26%) | 0.03 | 97.53 | 93.35 | 46.35 |
| BF1-S2-3 | 76252882 | 72506758 | 70727785(97.55%) | 0.03 | 97.68 | 93.55 | 46.22 |
| BF1-S3-1 | 58496470 | 56795488 | 55479363(97.68%) | 0.03 | 97.57 | 93.29 | 45.85 |
| BF1-S3-2 | 57466284 | 55563074 | 54212717(97.57%) | 0.03 | 97.3 | 92.71 | 46.14 |
| BF1-S3-3 | 73271944 | 68259610 | 66574090(97.53%) | 0.03 | 97.69 | 93.61 | 46.18 |
| HF1-S1-1 | 68516682 | 65500750 | 63807422(97.41%) | 0.03 | 97.57 | 93.41 | 46.41 |
| HF1-S1-2 | 53637572 | 51584960 | 50326829(97.56%) | 0.03 | 97.28 | 92.66 | 46.41 |
| HF1-S1-3 | 55763052 | 53582056 | 52317224(97.64%) | 0.03 | 97.44 | 93.09 | 46.43 |
| HF1-S2-1 | 64933282 | 62583986 | 61088943(97.61%) | 0.03 | 97.45 | 93 | 46.1 |
| HF1-S2-2 | 66306832 | 63013736 | 61530955(97.65%) | 0.03 | 97.39 | 92.97 | 46.09 |
| HF1-S2-3 | 71378618 | 68110396 | 66565232(97.73%) | 0.03 | 97.7 | 93.61 | 46.21 |
| HF1-S3-1 | 66223318 | 63464154 | 61955736(97.62%) | 0.03 | 97.41 | 92.9 | 45.98 |
| HF1-S3-2 | 70457626 | 67977502 | 66440076(97.74%) | 0.03 | 97.33 | 92.77 | 46.1 |
| HF1-S3-3 | 80033494 | 76516322 | 74767308(97.71%) | 0.03 | 97.68 | 93.56 | 46.01 |

**Supplementary Table 7.** DEGs of the BF1 and HF1 at different fruit development stages.

| Group name | Total | Up regulated | Down regulated |
| --- | --- | --- | --- |
| BF1-S1_vs_HF1-S1 | 643 | 426 | 217 |
| BF1-S2_vs_HF1-S2 | 428 | 191 | 237 |
| BF1-S3_vs_HF1-S3 | 755 | 209 | 546 |

**Supplementary Table 8.** DEGs related to carotenoid pathway in S1 stage.

| Gene | Gene ID | BF1-FPKM | HF1-FPKM | Log2FC | Regulated |
| --- | --- | --- | --- | --- | --- |
| *NCED* | Prupe.1G255500_v2.0.a1 | 31.14±0.03 | 4.6±0.16 | -2.78 | down |
| *CCD8* | Prupe.1G448400_v2.0.a1 | 0.90±0.10 | 0.33±0.06 | -1.44 | down |
| *ABA2* | Prupe.6G138700_v2.0.a1 | 0.17±0.02 | 1.95±0.38 | 3.51 | up |
| *ABA2* | Prupe.6G286600_v2.0.a1 | 0.05±0.02 | 1.12±0.09 | 4.46 | up |
| *ZEP* | Prupe.6G162100_v2.0.a1 | 3.06±0.21 | 7.51±0.06 | 1.28 | up |

**Supplementary Table 9.** DEGs related to carotenoid pathway in S2 stage.

| Gene | Gene ID | BF1-FPKM | HF1-FPKM | Log2FC | Regulated |
| --- | --- | --- | --- | --- | --- |
| *NCED* | Prupe.1G255500_v2.0.a1 | 57.91±0.35 | 6.06±0.29 | -3.28 | down |
| *CYP707A* | Prupe.5G013100_v2.0.a1 | 2.18±0.09 | 0.97±0.04 | -1.19 | down |

**Supplementary Table 10.** DEGs related to carotenoid pathway in S3 stage.

| Gene | Gene ID | BF1-FPKM | HF1-FPKM | Log2FC | Regulated |
| --- | --- | --- | --- | --- | --- |
| *NCED* | Prupe.1G255500_v2.0.a1 | 178.97±7.88 | 13.49±0.70 | -3.7 | down |
| *CYP707A* | Prupe.5G013100_v2.0.a1 | 1.62±0.11 | 0.66±0.12 | -1.27 | down |
