# Supplementary figures and images for: Down-regulation of NCED leads to the accumulation of carotenoids in the flesh of F1 generation of peach hybrid
Source: Front Plant Sci. 2022 Nov 3;13:1055779. doi: 10.3389/fpls.2022.1055779 (PMC9669654; doi:10.3389/fpls.2022.1055779)

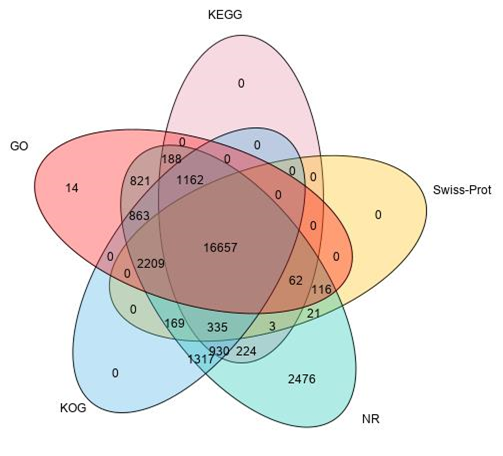

Supplement: Supplementary file 2 [file Image_1.tif]

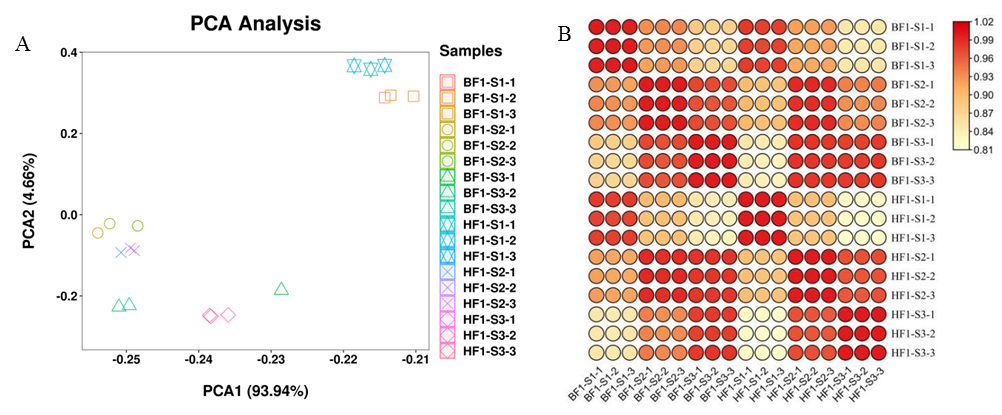

Supplement: Supplementary file 3 [file Image_2.tif]

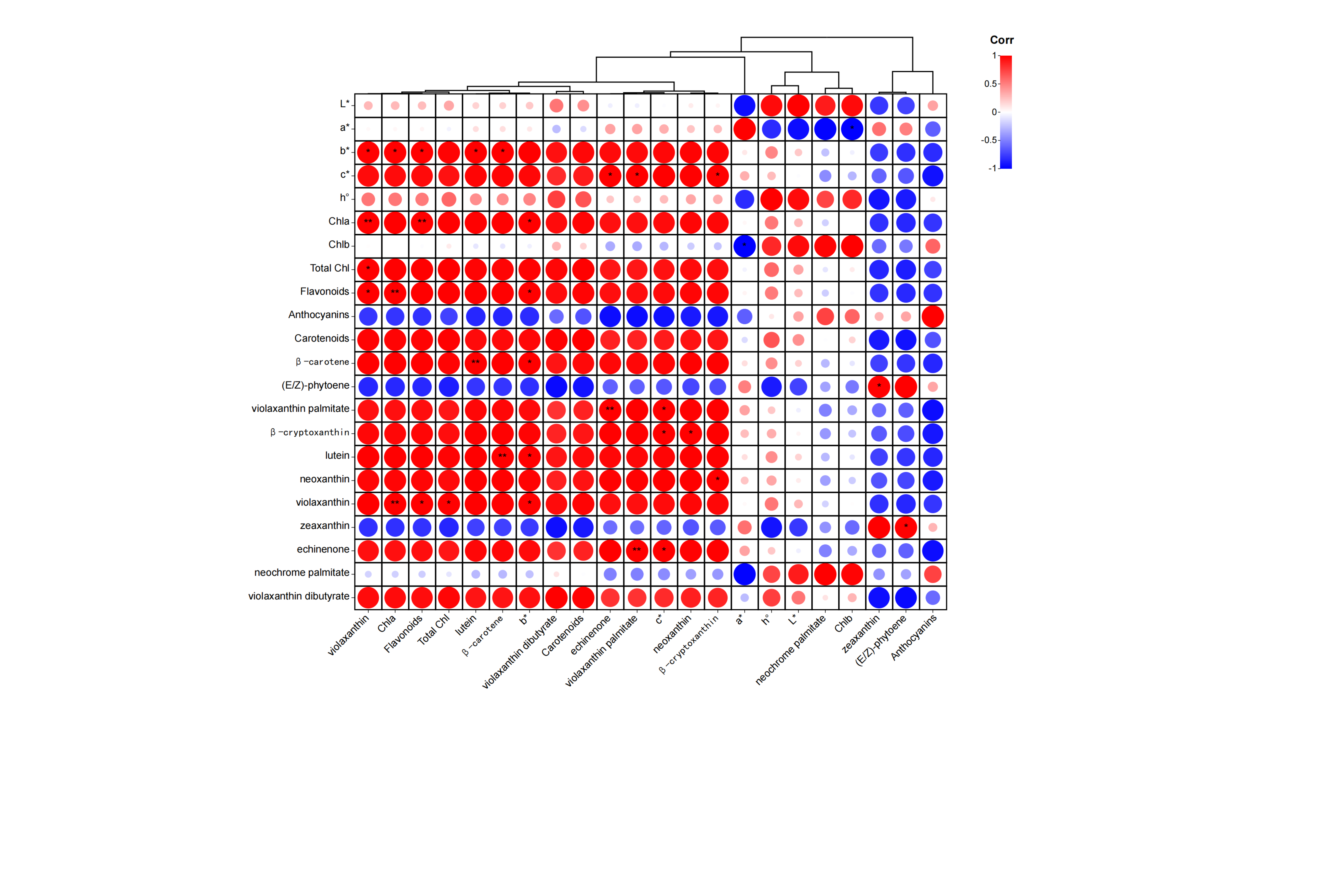

Supplement: Supplementary file 4 [file Image_3.tiff]
